# Propensity score matching comparing short-term nerve electrical stimulation to pulsed radiofrequency for herpes zoster-associated pain: A retrospective study

**DOI:** 10.3389/fnmol.2022.1069058

**Published:** 2022-11-28

**Authors:** Liu Liu, Wen-jing Zhang, Shu-xiang Xu, Wen-shuai Guo, Ran-ran Yan, Xiao-han Jiang, Shi-yao Li, Tao Sun

**Affiliations:** ^1^Department of Pain Management, Shandong Provincial Hospital, Shandong University, Jinan, China; ^2^Department of Pain Management, Shandong Provincial Hospital Affiliated to Shandong First Medical University, Jinan, China

**Keywords:** acute herpetic neuralgia, postherpetic neuralgia, pulsed radiofrequency, subacute herpetic neuralgia, zoster-associated pain, short-term nerve electrical stimulation

## Abstract

**Background:**

Zoster-associated pain (ZAP) is notoriously difficult to treat. Pulsed radiofrequency (PRF) and short-term nerve electrical stimulation (st-NES) have been proven effective treatments for ZAP. However, it is still unclear which technique provides improved analgesia in ZAP. This study is based on a large-scale, long-term follow-up to evaluate the efficacy and safety between st-NES and PRF.

**Materials and methods:**

All eligible ZAP patients treated with st-NES or PRF in our department were enrolled. Cohorts were divided into the st-NES group and the PRF group. A 1:1 ratio propensity score matching (PSM) was used to balance the baseline characteristics. The PS-matched cohort was adopted to investigate the efficacy and safety of the two treatments. The ordinal regression analysis was performed to determine the variables affecting the treatment effect of ZAP.

**Results:**

A total of 226 patients were included after PSM. The numerical rating scale (NRS) scores in st-NES and PRF groups considerably reduced compared to baseline levels after treatment. The NRS scores in the st-NES group were obviously lower than those in the PRF group at discharge, 1, 3, 6, 12, and 24 months. During the follow-up period, the NRS reduction rate remained higher in the st-NES group than in the PRF group (*P* < 0.01). The dosage of medication, Pittsburgh Sleep Quality Index (PSQI) score, and the number of patients with aggravated pain after discharge in the st-NES group were significantly less than in the PRF group after treatment.

**Conclusion:**

Short-term nerve electrical stimulation has been shown to be more advantageous than PRF for pain relief and quality of life improvement for ZAP patients.

## Introduction

Zoster-associated pain (ZAP) is common and difficult to treat (Forbes et al., 2016). Evidence suggested that 15% to –45% of patients with shingles subsequently progress to postherpetic neuralgia (PHN), especially in those over 60 years of age (Gershon et al., 2015; Moshayedi et al., 2018; Salvetti et al., 2019; Zhou J. et al., 2021). The generally accepted classification of ZAP is as follows: (i) acute herpetic neuralgia (AHN) within 30 days of onset, (ii) subacute herpetic neuralgia (SHN), pain recorded between 30 and 90 days, (iii) postherpetic neuralgia (PHN), defined as pain lasting more than 90 days after the presentation (Whitley et al., 2010).

Currently, the treatment for ZAP is primarily based on symptom control (van Wijck et al., 2006; Johnson and Rice, 2014). Medications are the most fundamental and principal management for ZAP, with pregabalin and gabapentin being the most commonly used first-line therapeutic agents (Finnerup et al., 2021). However, complete pain relief cannot be achieved by taking medications, and 20–40% of patients do not respond to the drugs (Binder and Baron, 2016). Conservative treatments such as acupuncture and physical therapy combined with pharmacy have been reported to provide greater pain relief for ZAP (Fleckenstein et al., 2009; Zhou Q. et al., 2021). However, these therapeutic effects are still limited and lack quality supporting evidence. Invasive treatments, including nerve blocks and neuromodulation, provide alternatives for ZAP patients who are not satisfied with the results of drugs and conservative treatments (Makharita and Amr, 2020). It has been reported that local anesthetics and steroids injections could alleviate AHN (van Wijck et al., 2006), while the therapeutic effects of PHN were usually unsatisfactory and disappointing. Neuromodulations, including PRF and NES, have been rapidly developed in the management of chronic pain over the past 20 years (Liu et al., 2020; Knotkova et al., 2021). NES includes st-NES and permanent NES. Although permanent NES can achieve prolonged analgesia, its application is limited by the high costs and high incidence of complications (Kumar et al., 2006; Deer et al., 2014). Hence, st-NES have been increasingly used for intractable pain including ZAP. Recent reports have indicated that PRF and st-NES are effective treatments for ZAP (Johnson and Burchiel, 2004; Yanamoto and Murakawa, 2012; Dong et al., 2017; Kim et al., 2017; Li et al., 2018; Makharita et al., 2018; Huang et al., 2020). However, it is still unclear which technique provides improved analgesia in ZAP. Previously published studies comparing the efficacy of st-NES and PRF for ZAP are inconsistent (Liu et al., 2020; Song, 2021; Sheng et al., 2022).

Therefore, we compared st-NES with PRF in the effectiveness of treatment for ZAP through PSM and investigated the factors that influenced the therapeutic effects of ZAP. We have further explored the efficacy of these two treatments in comparison to each other in terms of disease course and location. NRS score and the NRS reduction rate were selected as the primary endpoints. The secondary endpoints included medication consumption, PSQI score, the aggravation of pain, and side effects.

## Materials and methods

### Study design

This retrospective study was approved by the Ethics Committee of the Shandong Provincial Hospital. Inclusion criteria were as follows: (1) the patient’s clinical data was integrity, (2) ZAP with a precise diagnosis, (3) pre-operative NRS ≥ 4, (4) resistance to pharmacological treatment or intolerable side effects of drugs, and (5) stimulation treatment for 7–10 days. Exclusion criteria were as follows: (1) companies with other chronic pain in the same site with ZAP, (2) invasive treatment for ZAP within 2 weeks before this admission, (3) stimulation treatment for less than 7 days, and (4) patients with lost follow-up.

### Study population

**FIGURE 1:**
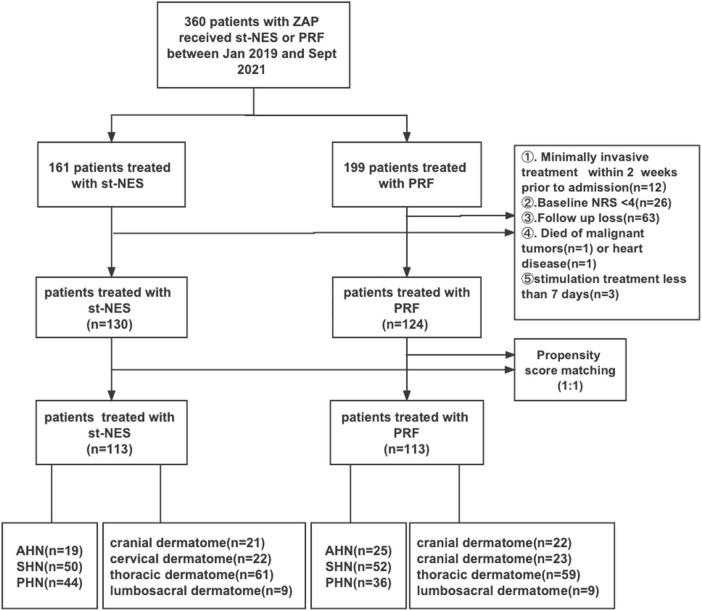
Study design and procedure.

As shown in Figure 1, Between January 2019 and September 2021, 360 consecutive patients with ZAP were hospitalized in our department receiving PRF or st-NES. A total of 254 patients were ultimately enrolled in the current study (st-NES group, *n* = 130 and PRF group, *n* = 124). The following were excluded from the study: lost to follow-up (*n* = 63), accepting invasive treatment within 2 weeks before admission (*n* = 12), baseline NRS < 4 (*n* = 26), and died of malignant tumors (*n* = 1) or heart disease (*n* = 1), stimulation treatment for less than 7 days (*n* = 3). After PSM, 113 patients were included in each group. The PS-matched cohort was further divided into different subgroups according to disease duration (19 AHN patients, 50 SHN patients, and 44 PHN patients in the st-NES group and 25 AHN patients, 52 SHN patients, and 36 PHN patients in the PRF group) and sites (st-NES group: cranial dermatome *n* = 21, cervical dermatome *n* = 22, thoracic dermatome *n* = 61, lumbosacral dermatome *n* = 9. PRF group: cranial dermatome *n* = 22, cervical dermatome *n* = 23, thoracic dermatome *n* = 59, lumbosacral dermatome *n* = 9).

### Surgical procedures

The procedure of st-NES was as follows: Short-term peripheral nerve stimulation (st-PNS) was applied in patients with ZAP in the cranial dermatome, short-term spinal cord stimulation (st-SCS) was applied in the cervical, thoracic or lumbosacral dermatome. In this study, st-PNS included supraorbital nerve and gasserian ganglion stimulation. X-ray guided exposure of the optimal puncture site and locating the target position of the electrode. The electrode was implanted through a puncture needle at a proper physiologic and anatomic position with local or general anesthesia. For supraorbital nerve stimulation, the supraorbital notch and supraorbital rim were located, slowly local infiltrate anesthesia with 1% lidocaine was performed from the medial edge of the contralateral brow arch to the superior edge of the affected brow arch to ensure that the supraorbital nerve was not blocked, then placed the puncture needle and reached the periosteal surface. The anterioposterior film showed that the puncture needle was located approximately 1 cm from the superior orbital rim of the affected side (Vaisman et al., 2012; Wan and Song, 2021). For gasserian ganglion stimulation, the oval foramen on the affected side was exposed as the puncture target, and the puncture needle was inserted in the established direction under general anesthesia. The direction and depth of the needle were adjusted under fluoroscopy. The electrode was placed after the needle tip passed through the oval fossa until the upper end of the electrode was shown to reach the junction between the sella turcica, the clivus, and the petrous part of the temporal bone on the lateral fluoroscopy. For spinal cord electrical stimulation, the puncture point was usually 1–2 segments below the target vertebral segment, and the electrode was located in the epidural space on lateral film (Taub et al., 1997). Then, Electrical stimulation was performed, and the electrode position and parameters (frequency, pulse width, and voltage) were adjusted to ensure adequate paresthesia caused by the stimulus could cover the painful area entirely. Then the position of the electrode was considered appropriate, and a fixation suture was applied to it (Song et al., 2014). The specific parameters were set according to the patient’s sensation, with frequencies mainly in the range of 60–100 Hz and without exceeding 300, pulse width usually between 90 and 300 us and no more than 1,000 us, and a voltage mainly varied from 0.5 to 5.5 V. In addition, the stimulus electrode and electric pulse generator were Medtronic (1*8 compact 3878-75, Medtronic, Minneapolis, MN, United States), and all patients were placed with a single electrode. The stimulation was continuous and performed for 7–10 days, and then the electrode was removed after the treatment. During the treatment, the parameters of the electrode were constantly adjusted to ensure that pain could be replaced entirely by pleasant paresthesia.

The targets of PRF in this study included the supraorbital nerve, the gasserian ganglion, and the dorsal root ganglion. The procedure of PRF is as follows: the anatomical area of the involved nerve was found under X-ray or ultrasound guidance and punctured with a radiofrequency needle with local anesthesia until the tip of the needle reached a satisfactory position. The supraorbital foramen was the puncture point for the supraorbital nerve PRF, the PRF needle was inserted perpendicular to the skin, and after the sense of falling, the PRF needle core was placed (Zhang et al., 2020). Similarly, the foramen ovale was the puncture target of the gasserian ganglion radiofrequency, and the puncture needle was inserted until it reached the foramen ovale. Then the tip of the needle was adjusted while inserting the needle core until the tip of the needle reached the clivus in the lateral fluoroscopy (Ding et al., 2019). For dorsal root ganglion PRF, the target vertebral segment was positioned as the puncture point and punctured under fluoroscopy or ultrasound until the needle tip was above or below the intervertebral foramen. After back drawing without blood or fluid, a stimulation test was performed under local anesthesia (Geurts et al., 2003). Sensory stimulation at a voltage of 0.2–0.6 V and a frequency of 50 Hz was implemented to ensure the needle tip position was closer to the surface of the ganglion or nerve. Subsequently, the parameters and their values were as follows: pulse width 15 or 20 ms, temperature 42°C, frequency 2 Hz, voltage 40–70 V, duration 600 s. Patients were usually treated with PRF 2–3 times during hospitalization, depending on the level of pain relief. If the pain was not relieved within 3 days after the treatment or the result was unsatisfactory, a further PRF treatment would be performed.

### Measurements

The primary outcomes include the numerical rating scale (NRS) and NRS reduction rate. NRS reduction rate was calculated as another indicator to assess the pain relief level of the treatment (Excellent: NRS reduction ≥80%, Medium: 50% ≤ NRS reduction <80%, Poor: NRS reduction <50%) (Rigoard et al., 2019, 2021). NRS score and NRS reduction rate were evaluated at baseline (pre-operative), discharge (post-operative), 1, 3, 6, 12, and 24 months after discharge.


$$NRSreductionrate\left(\%\right)=\frac{\begin{array}{c} (NRSscoreateachtimepoint\\ -baselineNRSscore) \end{array}}{b\,a\,s\,e\,l\,i\,n\,e\,N\,R\,S\,s\,c\,o\,r\,e}*100$$

The Pittsburgh Sleep Quality Index (PSQI), analgesic consumption, and the aggravation of pain after discharge were used as secondary outcome indicators. The PSQI was recorded at baseline, discharge, and final follow-up. Analgesic consumption (pregabalin or gabapentin) was recorded at pre-operative, 1, 3, 6, 12, and 24 months after discharge. the aggravation of pain and adverse effects were assessed post-operatively.

### Propensity score matching analysis

Propensity score matching (PSM) analysis was utilized to restrain confounding factors and settle possible patient selection bias. The PSM was based on age, side, duration of ZAP, and base NRS. Therefore, rigorous adjustment was implemented using nearest neighbor matching without replacement and the caliper width of 0.1 for significant differences in the underlying characteristics of PSM patients. After PSM, a *P*-value above 0.05 indicated a significant imbalance between groups.

### Statistical analysis

All data were analyzed using IBM SPSS Statistics 26.0 (SPSS IBM Corporation, Chicago, IL, USA). Continuous data are expressed as the mean ± standard error of the mean (x ± SEM), and the enumeration data are presented as numbers and proportions. Comparisons between two groups were performed using the chi-square test, independent samples *t*-test, and Mann–Whitney *U*-test, and appropriate statistical methods were selected based on the type of variables and whether they conformed to a normal distribution. Univariate χ^2^ analysis was first performed in analyzing prognostic factors. Afterward, the factor with *P* < 0.1 in the results of the univariate analysis was used as an independent variable entered the Ordinal regression analysis. At the same time, the odds ratio (OR) and its 95% confidence interval (CI) were calculated. *P* < 0.05 was deemed statistically significant.

## Results

### General characteristics

The general characteristics of the patients are summarized in Table 1. The differences in baseline information between the two groups were statistically significant before PSM (*P* = 0.008–0.048, Table 1). We matched the two groups according to a sample size of 1:1, and all basic characteristics increased to *P* > 0.05 between the groups after PSM.

**TABLE 1:** Patients’ characteristics.

	Before PSM				After PSM			
	st-NES (*n* = 130)	PRF (*n* = 124)	*X*^2^/t	*P*	St-NES (*n* = 113)	PRF (*n* = 113)	*X*^2^/t	*P*
Age (Y, x^–^ ± SEM)	68.6 ± 0.8	65.9 ± 0.8	−2.304	0.022*	68.5 ± 0.9	66.19 ± 0.9	1.811	0.071
Sex (*n*, %) M F	67 (51.5%) 63 (48.5%)	56 (45.2%) 68 (54.8%)	1.033	0.309	58 (51.3%) 55 (48.7%)	54 (47.8%) 59 (52.2%)	0.283	0.595
Side (*n*, %) L R	80 (61.5%) 50 (38.5%)	61 (49.2%) 63 (50.8%)	3.916	0.048*	68 (60.2%) 45 (39.8%)	60 (53.1%) 53 (46.9%)	1.153	0.283
Location (*n*, %) F C T L	23 (17.7%) 23 (17.7%) 73 (56.2%) 11 (8.3%)	23 (18.5%) 24 (19.4%) 67 (54%) 10 (8.1%)	0.184	0.98	21 (18.6%) 22 (19.5%) 61 (54.0%) 9 (8.0%)	22 (19.5%) 23 (20.4%) 59 (52.2%) 9 (8.0%)	0.079	0.994
BMI (x^–^ ± SEM)	24.4 ± 0.3	24.3 ± 0.3	−0.196	0.845	24.5 ± 0.3	24.2 ± 0.4	0.491	0624
Period (*n*, %) AHN SHN PHN	19 (14.6%) 53 (40.8%) 58 (44.6%)	35 (28.2%) 53 (42.7%) 36 (29%)	9.753	0.008*	19 (16.8%) 50 (44.2%) 44 (38.9%)	30 (22.1%) 43 (46.0%) 36 (31.9%)	1.657	0.437
Base NRS (*n*, %) 4–6 7–10	20 (15.4%) 110 (84.6%)	32 (25.8%) 92 (74.2%)	4.234	0.040*	18 (15.9%) 95 (84.1%)	28 (24.8%) 85 (75.2%)	2.273	0.099
Base PSQI (x^–^ ± SEM)	13.8 ± 0.2	13.4 ± 0.2	1.762	0.184	13.8 ± 0.2	13.4 ± 0.2	1.347	0.179
Hypertension history (YES/NO, %)	52 (40%) 78 (60%)	41 (33.1%) 83 (66.9%)	1.315	0.251	46 (40.7%) 67 (59.3%)	37 (32.7%) 76 (67.3%)	1.542	0.214
Coronary heart disease (YES/NO, %)	19 (14.6%) 111 (85.4%)	16 (12.9%) 108 (87.1%)	0.157	0.692	19 (16.8%) 94 (83.2%)	15 (13.3%) 98 (86.7%)	0.554	0.457
Diabetes history (YES/NO, %)	24 (17.7%) 107 (82.3%)	28 (22.6%) 96 (77.4%)	0.945	0.331	22 (19.5%) 91 (80.5%)	26 (23.0%) 87 (77.0%)	0.423	0.515

F, cranial dermatome; C, cervical dermatome; T, thoracic dermatome; L, lumbosacral dermatome, *P < 0.05, *P*-values for group comparisons by chi-square test or independent samples *t*-test.

### Comparison of the efficacy of the two groups

#### Primary endpoints

##### Pain relief

**FIGURE 2:**
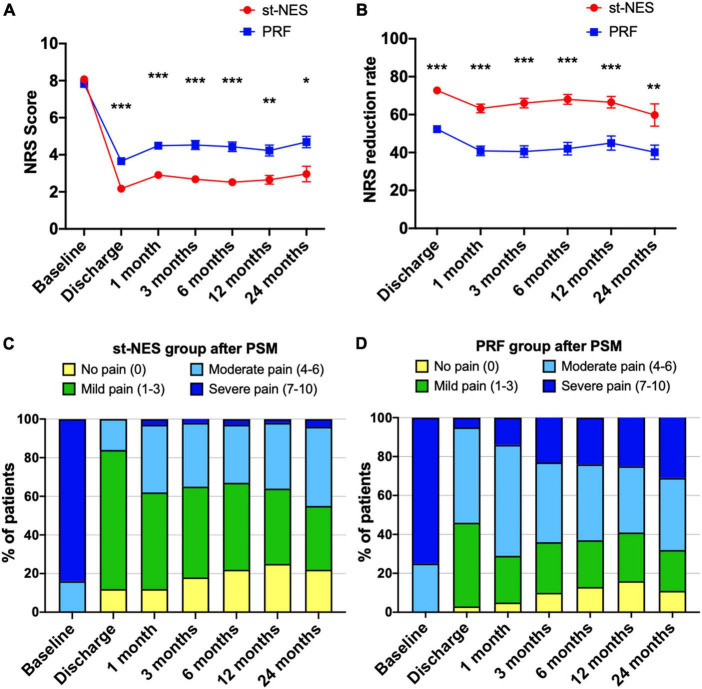
**(A,B)** Comparison of the efficacy of the two groups on pain relief after propensity score matching (PSM) by numerical rating scale (NRS) score or NRS reduction rate [*n* = 226, *P < 0.05, **P < 0.01,***P < 0.001, indicate pulsed radiofrequency (PRF) group vs. short-term nerve electrical stimulation (st-NES) group, *P*-values for group comparison by Mann–Whitney *U*-test]. **(C,D)** Percentage of patients with different pain levels in the st-NES group or PRF group.

The baseline average NRS score for the st-NES group was 8.1 ± 0.1, and for the PRF group, it was 7.8 ± 0.2, which were significantly reduced to 2.2 ± 0.3 and 3.7 ± 0.2 at discharge, respectively. Low pain scores were sustained at 1-month to 24-month follow-ups. NRS score in the st-NES group was reported to be significantly lower than in the PRF group at each time point after treatment (*P* < 0.01, Figure 2A). During the follow-up period, the NRS reduction rate remained higher in the st-NES group than in the PRF group (*P* < 0.01, Figure 2B). Additionally, 55–84% of patients achieved an NRS score ≤3 during follow-up in the st-NES group (Figure 2C), and it was 29–46% in the PRF group (Figure 2D).

##### The percentage of patients with different outcomes of pain relief

**FIGURE 3:**
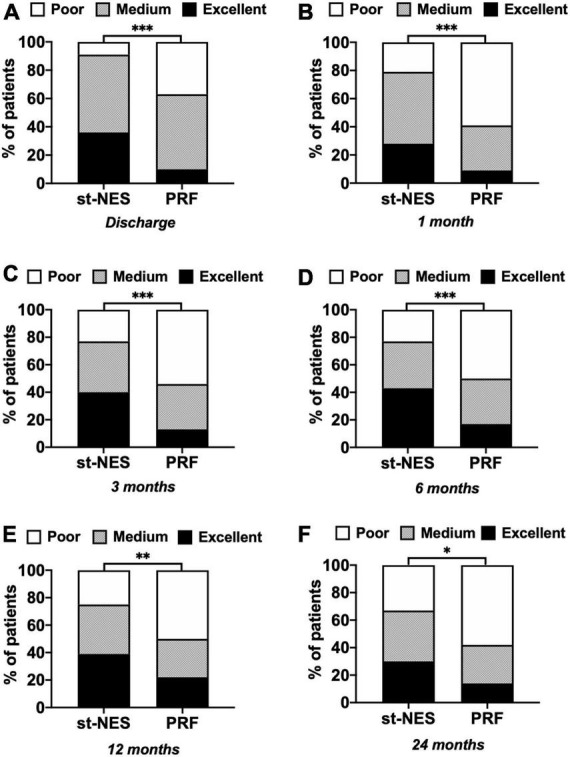
**(A–F)** The proportion of people with excellent, medium, and poor outcomes of numerical rating scale (NRS) reduction rate in short-term nerve electrical stimulation (st-NES) and pulsed radiofrequency (PRF) group at discharge, 1, 3, 6, 12, and 24 months follow-up (*n* = 226, *P < 0.05, **P < 0.01, ***P < 0.00, indicate PRF group vs. st-NES group, *P*-values for group comparison by chi-square test).

A total of 91% of patients at discharge, 77, and 67% of patients at 6 and 24 months had excellent and medium outcomes in the st-NES group. In the PRF group, patients with excellent and medium outcomes at discharge, 6 and 24 months were 63, 50, and 42%, respectively (1–6 months *P* < 0.0001 Figures 3A–D, 12 and 24 months *P* = 0.002, *P* = 0.019, Figures 3E,F).

##### Comparison in different disease duration

**FIGURE 4:**
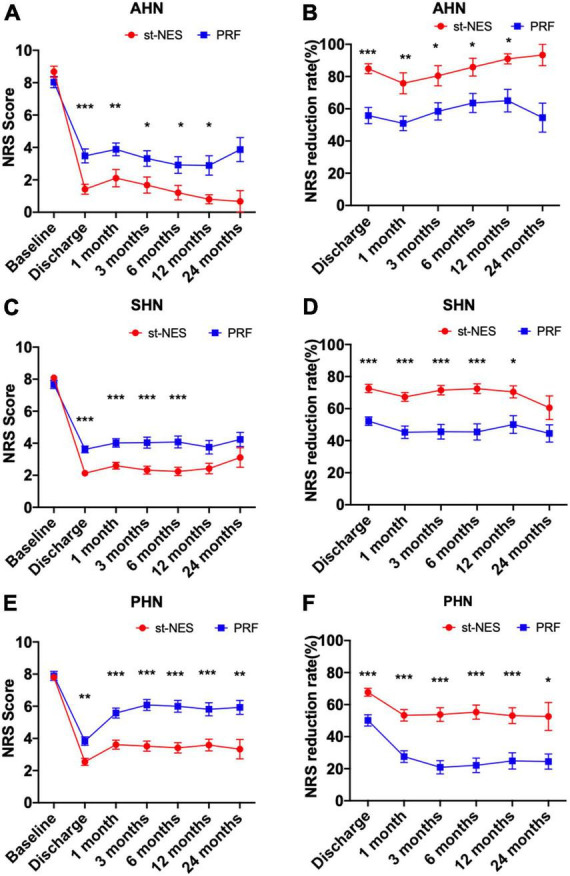
**(A–F)** Comparison of two treatment modalities for changes of numerical rating scale (NRS) score and NRS reduction rate at each time point during follow-up in acute herpetic neuralgia (AHN), subacute herpetic neuralgia (SHN), and postherpetic neuralgia (PHN), respectively [*n* = 226, *P < 0.05, **P < 0.01, ***P < 0.001, indicate pulsed radiofrequency (PRF) group vs. short-term nerve electrical stimulation (st-NES) group, *P*-values for group comparisons by Mann–Whitney *U*-test].

For AHN and SHN, the pain was significantly relieved with st-NES therapy than PRF therapy within 12 months, as seen by the lower NRS scores and higher NRS reduction rate (*P* = 0.0001–0.031 Figures 4A–D). No significant difference in NRS scores and NRS reduction rate was observed between the two groups after 12 months. For PHN, the average NRS score in the st-NES group was significantly reduced from 7.8 ± 0.2 to 2.5 ± 0.2 at discharge and remained between 3.3 ± 0.6 and 3.6 ± 0.3 from 1 to 24 months, that in the PRF group reduced from 7.9 ± 0.3 to 3.8 ± 0.3 and remained between 5.6 ± 0.3 and 6.1 ± 0.3 (*P* < 0.05, Figures 4E–F).

##### Comparison in different lesion sites

**FIGURE 5:**
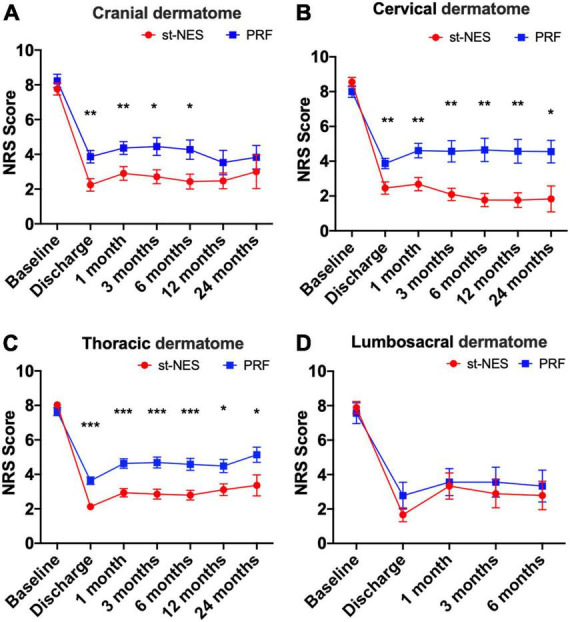
**(A–D)** Comparison of two groups treatment modalities at different sites [*n* = 226, *P < 0.05, **P < 0.01, ***P < 0.001, indicate pulsed radiofrequency (PRF) group vs. short-term nerve electrical stimulation (st-NES) group, *P*-values for group comparisons by Mann–Whitney *U*-test].

After treatments, NRS scores significantly declined in both groups at each time for ZAP occurring in the cranial, cervical, thoracic, and lumbosacral dermatome. However, in the cranial dermatome, NRS scores considerably decreased at discharge, 1, 3, and 6 months in the st-NES group compared with the PRF group (*P* ≤ 0.021, Figure 5A), there was no significant difference at 12 and 24 months. In the cervical dermatome and thoracic dermatome, compared with the NRS scores in the PRF group, the NRS scores in the st-NES group obviously declined at any follow-up interval (*P* ≤ 0.023, *P* ≤ 0.035, Figures 5B,C). Additionally, in the lumbosacral dermatome, there was no significant difference in NRS scores at each follow-up time in the two groups (*P* > 0.05, Figure 5D).

In addition, the operation method of peripheral nerve stimulation and pulsed radiofrequency therapy for herpetic neuralgia in the cranial dermatome will affect the curative effect. We divided the target nerves of peripheral nerve modulation into the supraorbital nerve region and the gasserian ganglion region and compared the efficacy of peripheral nerve electrical stimulation and radiofrequency in the same regions. As shown in Supplementary Figure 1, the NRS scores for supraorbital nerve electrical stimulation were significantly lower than supraorbital nerve radiofrequency within 6 months after discharge (Supplementary Figure 1A), while the efficacy of both was comparable after 6 months. The effects of gasserian ganglion stimulation and gasserian ganglion radiofrequency are always comparable (Supplementary Figure 1B).

#### Secondary endpoints

##### Medication consumption

**FIGURE 6:**
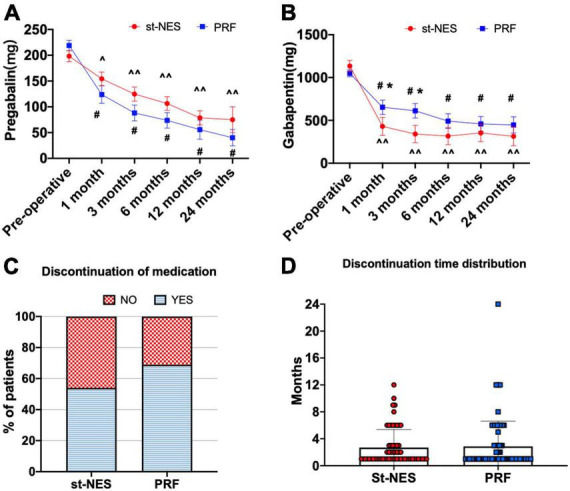
**(A,B)** The dosage of pregabalin and gabapentin before and after treatment [^∧^*P* < 0.05 and ^∧∧^*P* < 0.0001 indicate pre-operative vs. post-operative dosage in the short-term nerve electrical stimulation (st-NES) group, ^#^*P* < 0.0001 indicate pre-operative vs. post-operative dosage in the pulsed radiofrequency (PRF) group, *P < 0.05 indicate PRF group vs. st-NES group, *P*-values for inter- and intra-group comparisons by Mann–Whitney *U*-test]. **(C)** Proportion of patients who discontinued medication during the follow-up. **(D)** Distribution of discontinuation times and number of patients.

Compared to pre-operative dosages, the average dosages of pregabalin and gabapentin significantly decreased after treatment in the two groups post-operatively (*P* < 0.05, Figures 6A,B). There was no significant difference in the dosages of pregabalin at any time interval in the two groups, and the dosages of gabapentin obviously declined at 1- and 3-month in the st-NES group compared with the PRF group follow-up (*P* ≤ 0.033, Figure 6B). During the follow-up period, 54.0% of patients had stopped taking any analgesic within 12 months in the st-NES group and 69.0% in the PRF group (*P* = 0.067, Figures 6C,D).

##### Pittsburgh Sleep Quality Index and the aggravation of pain after discharge

**FIGURE 7:**
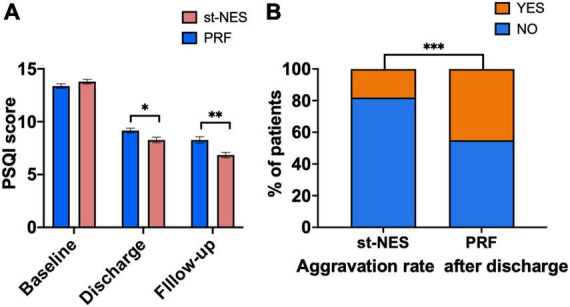
**(A)** Pittsburgh Sleep Quality Index (PSQI) scores decreased at discharge and final follow-up period. **(B)** Aggravation rates of the two groups (*n* = 226, *P < 0.05, **P < 0.01, ***P < 0.001, *P*-values for group comparison by Mann–Whitney *U*-test).

As shown in Figure 7A, the average PSQI scores in the two groups declined at discharge, which further declined at the end of follow-up. Compared to baseline, the average scores in the st-NES group decreased by 5.5 points at discharge and 7.0 points at the end of follow-up, which decreased more than those in the PRF group (*P* ≤ 0.001, Figure 7A). The number of people with pain worse after discharge than at discharge was significantly higher in the PRF group than in the st-NES group (*P* < 0.0001, Figure 7B). A total of 45.1% of patients in the PRF group and 17.7% in the st-NES group experienced an aggravation of pain within 6 months after discharge (*P* < 0.0001, Figure 7B).

#### Side effects

One patient developed leakage of cerebrospinal fluid, and two patients developed a local infection of the puncture sites in the st-NES group. These complications resulted in electrodes being removed prematurely for less than 10 days and disappearing after rehydration and administration of antibiotics. No other serious adverse events (including prolonged bleeding, hematoma formation, spinal cord injury, etc.) were found in the two groups.

### Influential factors

The cohort before PSM was used for risk factors analysis. Coronary heart disease, therapies, and disease duration may affect the effectiveness of ZAP treatment according to the results of the univariate analysis (*P* = 0.012, *P* < 0.0001, *P* < 0.0001, Table 2). Multivariable analysis showed that the therapeutic effect in the st-NES group was more significant than those in the PRF group (OR:5.417, 95% CI: 3.187–9.207, *P* < 0.0001, Table 2), and patients with shorter duration of disease were prone to achieve more excellent therapeutic results (AHN vs. PHN OR:8.784, 95% CI: 4.256–18.133, *P* < 0.0001, SHN vs. PHN OR:3.767, 95% CI: 4.256–18.133, *P* < 0.0001, Table 2).

**TABLE 2:** Risk factors for efficacy after minimally invasive neuromodulation (*n* = 254).

	Univariate analysis (*X*^2^)	*P*-value	Multivariate analysis OR (95% CI)	*P*-value
Sex	4.689	0.321		
Side	2.110	0.348		
Base NRS	1.214	0.545		
Hypertension	1.467	0.480		
Diabetes disease	2.023	0.364		
Coronary heart disease	8.838	0.012*	2.036 (0.986–4.206)	0.055
Age	0.757	0.685		
BMI	0.491	0.782		
Therapy	25.359	<0.0001*		
st-NES (vs. PRF)			5.417 (3.187−9.207)	<0.0001*
Disease duration	25.026	<0.0001*		
AHN (vs. PHN)			8.784 (4.256–18.133)	<0.0001*
SHN (vs. PHN)			3.767 (2.122–6.687)	<0.0001*
Location (F/C/T/L)	6.769	0.343		

Data are shown as chi-square values and odds ratio (95% confidence interval). The χ^2^ test was used for univariate analysis. The ordinal logistic regression was used for multivariate analyses of factors having *P* < 0.10 during univariate analysis. *P < 0.05.

## Discussion

Renovative varicella-zoster virus (VZV) leads to extensive necrosis of skin and nerve cells and triggers abnormal action of neurons resulting in ZAP (Wall and Gutnick, 1974; Devor, 1991; Head et al., 1997). Currently, a proportion of patients still turned PHN after performing interventions in the acute and subacute phases. PHN is the most common complication of herpes zoster and has produced moderate to severe pain for years, severely affecting the quality of life of patients and their families (Johnson, 2010). PRF and NES offer alternatives when conservative treatment and medication for intractable and refractory pain are not effective (Kemler et al., 2000; Eyigor et al., 2010; Assaf et al., 2016).

At present, PRF and st-NES, including st-SCS and st-PNS, were reported to be safe and effective for ZAP (Yanamoto and Murakawa, 2012; Ke et al., 2013; Dong et al., 2017; Wu et al., 2020; Wan and Song, 2021). However, there are relatively few studies on st-NES for treating ZAP compared to PRF, and the comparative efficacy between st-NES and PRF for ZAP is unclear. The present study was designed to determine the effect of st-NES and PRF in treating ZAP. We found that both PRF and st-NES could induce pain alleviation, and pain relief reported in patients treated with st-NES is higher than in those treated with PRF at any time interval. A previous study enrolled 91 AHN and SHN patients, followed up to 6 months, then observed that st-SCS achieved better pain relief than PRF for ZAP at 1-and 6-month follow-ups (Song, 2021). In addition, a recent study with a sample size of 70 PHN patients and a follow-up period of 12 months demonstrated that the VAS scores in the st-SCS group were reported notably lower than those in the PRF group at 3, 6, and 12 months after treatment (Sheng et al., 2022). Our findings are consistent with those studies. Furthermore, we observed that at 24 months follow-up, the st-NES group still maintained a lower NRS score and higher NRS reduction rate.

Apart from pain relief, medication consumption, PSQI score, and the aggravation of pain relevant to the quality of life were assessed in our study. We only counted the dosage of pregabalin and gabapentin due to irregular use of opioids, antidepressants, and other medications in patients. Consequently, a significant reduction in the dosage of these two drugs after surgery could be founded in the present study. Moreover, more than 50% of patients had stopped taking analgesic medication in both groups during the follow-up period. Most patients stopped taking medication gradually as the pain could be maintained at a lower level without a tendency to rebound. Lack of continuous sound sleep is widespread concern for patients with ZAP. In this study, PSQI scores decreased in the two groups, which observed in the st-NES group apparently declined compared to the PRF group. Additionally, patients treated with PRF were prone to have worse pain after discharge than at discharge compared to patients treated with st-NES. The results from secondary-endpoint assessments in the two groups further supported the NRS and NRS reduction rate analysis. Therefore, the superior long- and short-term efficacy of st-NES over PRF was derived from this observational study. This result may be explained by the fact that st-NES is continuous for 7–10 days while PRF lasts for 10 min once a time. The difference in the mechanism of these two treatment modalities may also be the cause. Prior studies reported that PRF could alternate the expression of inflammatory cytokines to induce analgesia, such as IL-6, IL-17, IFN-γ, TNFα, and IGF-2 (Das et al., 2018; Sam et al., 2021). Expression of the neurotransmitter GABA and the inhibitory GABAergic interneurons in the dorsal horn of the spinal cord and ganglion have been identified in a rat model as being involved in the mechanism of NES-mediated analgesia (Cui et al., 1997; Daniele and MacDermott, 2009; Takeda et al., 2013; Sun et al., 2018; Meuwissen et al., 2020). In addition, several reports have mentioned that electrical stimulation changes the electrical state of individual neurons, causing neurotransmitter activity, altering neuronal circuits, and leading to changes in pain and function (Gilmore et al., 2019; Sivanesan et al., 2019; Knotkova et al., 2021). Hence, the altered individual neurons and the neurotransmitters can still relieve pain by reducing the excitability of sensory neurons after the electrodes are removed.

Moreover, we further observed the comparative effects of the two treatments in different disease duration and sites, respectively. For AHN and SHN, the long-term efficacy observed in the st-NES group was comparable with the PRF group. However, st-NES was more effective than PRF for PHN in pain relief. A previous study reported no significant difference between the st-SCS and PRF within 24 weeks post-operatively in patients with SHN and PHN (Liu et al., 2020). The differences observed between us and that study may be due to differences in sample size and follow-up time. In the cervical and thoracic dermatome, the long and short-term efficacy of st-NES was greater than PRF. The statistical analysis did not include the NRS scores at 12 and 24 months in the lumbosacral dermatome because of the small sample size.

The influential factors associated with the therapeutic outcome of ZAP were evaluated. Ultimately, treatments and disease duration were influential factors in the efficacy of ZAP, which means that patients with st-NES experienced greater effectiveness than PRF, and the longer the course of the disease, the worse the outcome of the patients. This result differed from a previous study, which showed no effect of disease duration on the efficacy of ZAP (Liu et al., 2020). This inconsistency may result from the different disease duration of included patients between the two studies.

This study is real-world-based, where patients were admitted to the hospital for further treatment only when medication was ineffective or the side effects were intolerable. We have introduced PRF and st-NES to the patients in detail, and they chose the specific method. Therefore, each patient as their own control could indicate that st-NES and PRF were more effective than oral medications. However, several limitations of this study should be addressed in future research. First, this is a retrospective analysis, lacking strict randomized control. Thus we used propensity score matching methods to avoid the impacts of other variables on endpoints, and the obtained results were reviewed by various statistical methods to ensure the reliability of the data. Second, patients enrolled in one pain management center despite the large sample size. Future multiple centers studies are warranted to validate our findings.

## Conclusion

This study showed that st-NES and PRF are effective and safe in treating ZAP. St-NES provides better pain relief and sleeps improvement than PRF for ZAP patients. We further found that st-NES is more effective than PRF within 12 months in AHN, SHN patients, and patients with ZAP in the cranial dermatome, but the efficacy of st-NES and PRF is comparable after 12 months. There is no significant difference in the efficacy of the two treatment modalities for ZAP in the lumbosacral dermatome. In addition, the prognosis of ZAP is related to the type of treatment and duration of the disease, with no correlation to gender, age, underlying diseases, BMI, location, or side of the disease.

## Data availability statement

The raw data supporting the conclusions of this article will be made available by the authors, without undue reservation.

## Author contributions

LL contributed to writing of the manuscript. LL and W-JZ contributed to statistical analysis. LL, W-JZ, and TS contributed to study design. LL, W-JZ, S-XX, W-SG, R-RY, X-HJ, and S-YL contributed to collecting data. All authors contributed to the article and approved the submitted version.

## References

[B1] Assaf A. T. Hillerup S. Rostgaard J. Puche M. Blessmann M. Kohlmeier C. (2016). Technical and surgical aspects of the sphenopalatine ganglion (SPG) microstimulator insertion procedure. *Int. J. Oral. Maxillofac. Surg.* 45 245–254. 10.1016/j.ijom.2015.09.023 26559753

[B2] Binder A. Baron R. (2016). The pharmacological therapy of chronic neuropathic pain. *Dtsch. Arztebl. Int.* 113 616–625. 10.3238/arztebl.2016.0616 27697147 PMC5541246

[B3] Cui J. G. O’Connor W. T. Ungerstedt U. Linderoth B. Meyerson B. A. (1997). Spinal cord stimulation attenuates augmented dorsal horn release of excitatory amino acids in mononeuropathy via a GABAergic mechanism. *Pain* 73 87–95. 10.1016/s0304-3959(97)00077-8 9414060

[B4] Daniele C. A. MacDermott A. B. (2009). Low-threshold primary afferent drive onto GABAergic interneurons in the superficial dorsal horn of the mouse. *J. Neurosci.* 29 686–695. 10.1523/JNEUROSCI.5120-08.2009 19158295 PMC2826179

[B5] Das B. Conroy M. Moore D. Lysaght J. McCrory C. (2018). Human dorsal. root ganglion pulsed radiofrequency treatment modulates cerebrospinal fluid lymphocytes and neuroinflammatory markers in chronic radicular pain. *Brain Behav. Immun.* 70 157–165. 10.1016/j.bbi.2018.02.010 29458195

[B6] Deer T. R. Mekhail N. Provenzano D. Pope J. Krames E. Thomson S. (2014). The appropriate use of neurostimulation: Avoidance and treatment of complications of neurostimulation therapies for the treatment of chronic pain. *Neuromodulation* 17 571–598. 10.1111/ner.12206 25112891

[B7] Devor M. (1991). Neuropathic pain and injured nerve: Peripheral mechanisms. *Br. Med. Bull.* 47 619–630. 10.1093/oxfordjournals.bmb.a072496 1794075

[B8] Ding Y. Hong T. Li H. Yao P. Zhao G. (2019). Efficacy of CT guided pulsed radiofrequency treatment for trigeminal postherpetic neuralgia. *Front. Neurosci.* 13:708. 10.3389/fnins.2019.00708 31354417 PMC6630731

[B9] Dong D.-S. Yu X. Wan C.-F. Liu Y. Zhao L. Xi Q. (2017). Efficacy of. short-term spinal cord stimulation in acute/subacute zoster-related pain: A retrospective study. *Pain Physician* 20:E633–E645. 28727708

[B10] Eyigor C. Eyigor S. Korkmaz O. K. Uyar M. (2010). Intra-articular corticosteroid injections versus pulsed radiofrequency in painful shoulder: A prospective, randomized, single-blinded study. *Clin. J. Pain* 26 386–392. 10.1097/AJP.0b013e3181cf5981 20473045

[B11] Finnerup N. B. Kuner R. Jensen T. S. (2021). Neuropathic pain: From. mechanisms to treatment. *Physiol. Rev.* 101 259–301. 10.1152/physrev.00045.2019 32584191

[B12] Fleckenstein J. Kramer S. Hoffrogge P. Thoma S. Lang P. M. Lehmeyer L. (2009). Acupuncture in acute herpes zoster pain therapy (ACUZoster) - design and protocol of a randomised controlled trial. *BMC Complement Altern. Med.* 9:31. 10.1186/1472-6882-9-31 19674449 PMC2739152

[B13] Forbes H. J. Thomas S. L. Smeeth L. Clayton T. Farmer R. Bhaskaran K. (2016). A systematic review and meta-analysis of risk factors for postherpetic neuralgia. *Pain* 157 30–54. 10.1097/j.pain.0000000000000307 26218719 PMC4685754

[B14] Gershon A. A. Breuer J. Cohen J. I. Cohrs R. J. Gershon M. D. Gilden D. (2015). Varicella zoster virus infection. *Nat. Rev. Dis. Primers* 1:15016. 10.1038/nrdp.2015.16 27188665 PMC5381807

[B15] Geurts J. W. M. Knape J. T. A. Groen G. J. (2003). Radiofrequency lesioning. of dorsal root ganglia for chronic lumbosacral radicular pain: A randomised, double-blind, controlled trial. *Lancet* 361:6. 10.1016/s0140-6736(03)12115-0 12517462

[B16] Gilmore C. Ilfeld B. Rosenow J. Li S. Desai M. Hunter C. (2019). Percutaneous peripheral nerve stimulation for the treatment of chronic neuropathic postamputation pain: A multicenter, randomized, placebo-controlled trial. *Reg. Anesth. Pain Med.* 44 637–645. 10.1136/rapm-2018-100109 30954936

[B17] Head H. Campbell A. W. Kennedy P. G. (1997). The pathology of herpes. zoster and its bearing on sensory localisation. *Rev. Med. Virol.* 7 131–143. 10.1002/(sici)1099-1654(199709)7:3<131::aid-rmv198>3.0.co;2-7 10398478

[B18] Huang J. Yang S. Yang J. Sun W. Jiang C. Zhou J. (2020). Early treatment with temporary spinal cord stimulation effectively prevents development of postherpetic neuralgia. *Pain Physician* 23:E219–E230. 32214307

[B19] Johnson M. D. Burchiel K. J. (2004). Peripheral stimulation for treatment of. trigeminal postherpetic neuralgia and trigeminal posttraumatic neuropathic pain: A pilot study. *Neurosurgery* 55 135–141. 15214982

[B20] Johnson R. W. (2010). Herpes zoster and postherpetic neuralgia. *Expert Rev. Vaccines* 9 21–26. 10.1586/erv.10.30 20192714

[B21] Johnson R. W. Rice A. S. C. (2014). Postherpetic neuralgia. *N. Engl. J. Med.* 371 1526–1533. 10.1056/NEJMcp1403062 25317872

[B22] Ke M. Yinghui F. Yi J. Xeuhua H. Xiaoming L. Zhijun C. (2013). Efficacy. of pulsed radiofrequency in the treatment of thoracic postherpetic neuralgia from the angulus costae: A randomized, double-blinded, controlled trial. *Pain Physician* 16 15–25. 23340530

[B23] Kemler M. A. Barendse G. A. van Kleef M. de Vet H. C. Rijks C. P. Furnée C. A. (2000). Spinal cord stimulation in patients with chronic reflex sympathetic dystrophy. *N. Engl. J. Med.* 343 618–624. 10.1056/NEJM200008313430904 10965008

[B24] Kim K. Jo D. Kim E. (2017). Pulsed radiofrequency to the dorsal root. ganglion in acute herpes zoster and postherpetic neuralgia. *Pain Physician* 20:E411–E418. 28339440

[B25] Knotkova H. Hamani C. Sivanesan E. Le Beuffe M. F. E. Moon J. Y. Cohen S. P. (2021). Neuromodulation for chronic pain. *Lancet* 397 2111–2124. 10.1016/S0140-6736(21)00794-7 34062145

[B26] Kumar K. Wilson J. R. Taylor R. S. Gupta S. (2006). Complications of spinal. cord stimulation, suggestions to improve outcome, and financial impact. *J. Neurosurg. Spine* 5 191–203. 10.3171/spi.2006.5.3.191 16961079

[B27] Li D. Sun G. Sun H. Wang Y. Wang Z. Yang J. (2018). Combined therapy of pulsed radiofrequency and nerve block in postherpetic neuralgia patients: A randomized clinical trial. *PeerJ* 6:e4852. 10.7717/peerj.4852 29888123 PMC5991296

[B28] Liu B. Yang Y. Zhang Z. Wang H. Fan B. Sima L. (2020). Clinical study of spinal cord stimulation and pulsed radiofrequency for management of herpes zoster-related pain persisting beyond acute phase in elderly patients. *Pain Physician* 8 263–270. 32517392

[B29] Makharita M. Y. Amr Y. M. (2020). Effect of repeated paravertebral injections. with local anesthetics and steroids on prevention of post-herpetic neuralgia. *Pain Physician* 23 565–572. 33185373

[B30] Makharita M. Y. El Bendary H. M. Sonbul Z. M. Ahmed S. E. S. Latif M. A. (2018). Ultrasound-guided pulsed radiofrequency in the management of thoracic postherpetic neuralgia: A randomized, double-blinded, controlled trial. *Clin. J. Pain* 34 1017–1024. 10.1097/AJP.0000000000000629 29757758

[B31] Meuwissen K. P. V. de Vries L. E. Gu J. W. Zhang T. C. Joosten E. A. J. (2020). Burst and tonic spinal cord stimulation both activate spinal GABAergic mechanisms to attenuate pain in a rat model of chronic neuropathic pain. *Pain Pract.* 20 75–87. 10.1111/papr.12831 31424152 PMC7004135

[B32] Moshayedi P. Thomas D. Rinaldo C. R. Moossy J. J. Maroon J. C. Murdoch G. H. (2018). Subacute histopathological features in a case of varicella zoster virus myelitis and post-herpetic neuralgia. *Spinal Cord Ser. Cases* 4:33. 10.1038/s41394-018-0068-5 29707236 PMC5884843

[B33] Rigoard P. Basu S. Desai M. Taylor R. Annemans L. Tan Y. (2019). Multicolumn spinal cord stimulation for predominant back pain in failed back surgery syndrome patients: A multicenter randomized controlled trial. *Pain* 160 1410–1420. 10.1097/j.pain.0000000000001510 30720582 PMC6553955

[B34] Rigoard P. Roulaud M. Goudman L. Adjali N. Ounajim A. Voirin J. (2021). Comparison of spinal cord stimulation vs. dorsal root ganglion stimulation vs. association of both in patients with refractory chronic back and/or lower limb neuropathic pain: An international, prospective, randomized, double-blinded, crossover trial (BOOST-DRG Study). *Medicina* 58:7. 10.3390/medicina58010007 35056316 PMC8780129

[B35] Salvetti A. Ferrari V. Garofalo R. Gazzaniga P. Guerroni A. Metrucci A. (2019). Incidence of herpes zoster and postherpetic neuralgia in Italian adults aged ≥50 years: A prospective study. *Prev. Med. Rep.* 14:100882. 10.1016/j.pmedr.2019.100882 31193254 PMC6522697

[B36] Sam J. Catapano M. Sahni S. Ma F. Abd-Elsayed A. Visnjevac O. (2021). Pulsed radiofrequency in interventional pain management: Cellular and molecular mechanisms of action - an update and review. *Pain Physician* 24 525–532. 34793641

[B37] Sheng L. Liu Z. Zhou W. Li X. Wang X. Gong Q. (2022). Short-Term spinal. cord stimulation or pulsed radiofrequency for elderly patients with postherpetic neuralgia: A prospective randomized controlled trial. *Neural Plast.* 2022:7055697. 10.1155/2022/7055697 35529453 PMC9068337

[B38] Sivanesan E. Stephens K. E. Huang Q. Chen Z. Ford N. C. Duan W. (2019). Spinal cord stimulation prevents paclitaxel-induced mechanical and cold hypersensitivity and modulates spinal gene expression in rats. *Pain Rep.* 9:e785. 10.1097/PR9.0000000000000785 31875188 PMC6882571

[B39] Song J. J. Popescu A. Bell R. L. (2014). Present and potential use of spinal cord stimulation to control chronic pain. *Pain Physician* 17 235–246. 24850105

[B40] Song T. (2021). Efficacy of pulsed radiofrequency or short-termspinal cord. stimulation for acute/subacutezoster-related pain: A randomized, double-blinded, controlled trial. *Pain Phys.* 24 215–222. 10.36076/ppj.2021/24/215 33988940

[B41] Sun K.-F. Feng W.-W. Liu Y.-P. Dong Y.-B. Gao L. Yang H.-L. (2018). Electrical peripheral nerve stimulation relieves bone cancer pain by inducing arc protein expression in the spinal cord dorsal horn. *J. Pain Res.* 11 599–609. 10.2147/JPR.S149470 29606887 PMC5868598

[B42] Takeda M. Ikeda M. Takahashi M. Kanazawa T. Nasu M. Matsumoto S. (2013). Suppression of ATP-induced excitability in rat small-diameter trigeminal ganglion neurons by activation of GABAB receptor. *Brain Res. Bull.* 98 155–162. 10.1016/j.brainresbull.2013.08.005 24004472

[B43] Taub E. Munz M. Tasker R. R. (1997). Chronic electrical stimulation of the. gasserian ganglion for the relief of pain in a series of 34 patients. *J. Neurosurg.* 86 197–202. 10.3171/jns.1997.86.2.0197 9010419

[B44] Vaisman J. Markley H. Ordia J. Deer T. (2012). The treatment of medically. intractable trigeminal autonomic cephalalgia with supraorbital/supratrochlear stimulation: A retrospective case series. *Neuromodulation* 15 374–380. 10.1111/j.1525-1403.2012.00455.x 22551506

[B45] van Wijck A. J. M. Opstelten W. Moons K. G. M. van Essen G. A. Stolker R. J. Kalkman C. J. (2006). The PINE study of epidural steroids and local anaesthetics to prevent postherpetic neuralgia: A randomised controlled trial. *Lancet* 367 219–224. 10.1016/S0140-6736(06)68032-X 16427490

[B46] Wall P. D. Gutnick M. (1974). Properties of afferent nerve impulses originating from a neuroma. *Nature* 248 740–743. 10.1038/248740a0 4365049

[B47] Wan C.-F. Song T. (2021). Short-Term peripheral nerve stimulation relieve pain for elder herpes zoster ophthalmicus patients: A retrospective study. *Neuromodulation* 24 1121–1126. 10.1111/ner.13288 33058443 PMC8451917

[B48] Whitley R. J. Volpi A. McKendrick M. Wijck A. V. Oaklander A. L. (2010). Management of herpes zoster and post-herpetic neuralgia now and in the future. *J. Clin. Virol.* 48:S20–S28. 10.1016/S1386-6532(10)70005-6 20510264

[B49] Wu C.-Y. Lin H.-C. Chen S.-F. Chang W.-P. Wang C.-H. Tsai J.-C. (2020). Efficacy of pulsed radiofrequency in herpetic neuralgia: A meta-analysis of randomized controlled trials. *Clin. J. Pain* 36 887–895. 10.1097/AJP.0000000000000867 32701526

[B50] Yanamoto F. Murakawa K. (2012). The effects of temporary spinal cord. stimulation (or Spinal Nerve Root Stimulation) on the management of early postherpetic neuralgia from one to six months of its onset. *Neuromodulation* 15 151–154. 10.1111/j.1525-1403.2012.00438.x 22376181

[B51] Zhang H. Ni H. Liu S. Xie K. (2020). Supraorbital nerve radiofrequency for. severe neuralgia caused by herpes zoster ophthalmicus. *Pain Res. Manag.* 2020:3191782. 10.1155/2020/3191782 33062083 PMC7533012

[B52] Zhou J. Sun W. Liu Y. Yang S. Wu S. Wang S. (2021a). Clinical characteristics, treatment effectiveness, and predictors of response to pharmacotherapeutic interventions among patients with herpetic-related neuralgia: A retrospective analysis. *Pain Ther.* 10 1511–1522. 10.1007/s40122-021-00303-7 34510386 PMC8586103

[B53] Zhou Q. Wei S. Zhu H. Hu Y. Liu Y. Yang H. (2021b). Acupuncture and moxibustion combined with cupping for the treatment of post-herpetic neuralgia: A meta-analysis. *Medicine* 100:e26785. 10.1097/MD.0000000000026785 34397828 PMC8341313

