# Supplementary figures and images for: Propensity score matching comparing short-term nerve electrical stimulation to pulsed radiofrequency for herpes zoster-associated pain: A retrospective study
Source: Front Mol Neurosci. 2022 Nov 28;15:1069058. doi: 10.3389/fnmol.2022.1069058 (PMC9742239; doi:10.3389/fnmol.2022.1069058)

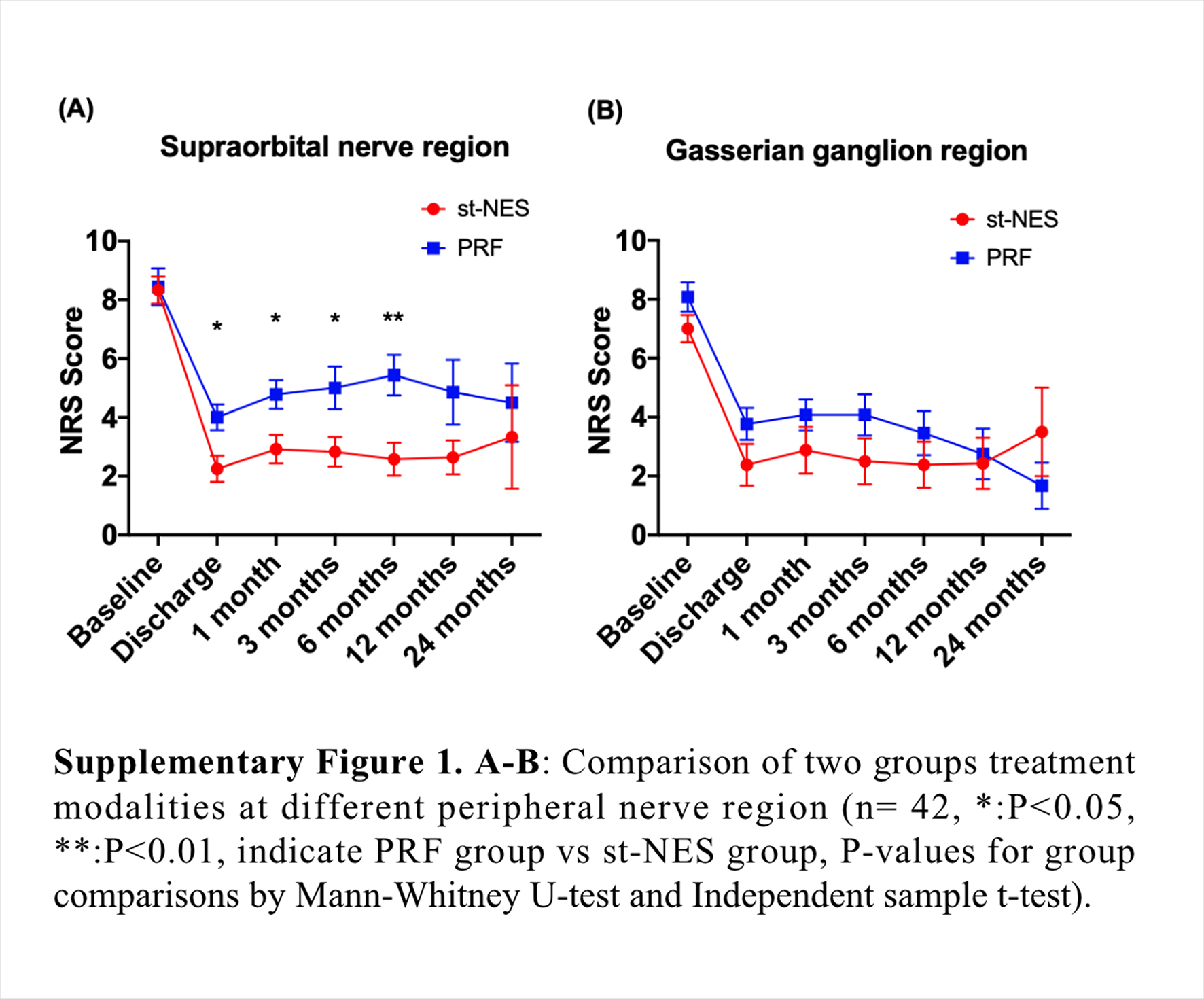

Supplement: Supplementary file 1 [file Image_1.tif]
